# Viral metagenomics of Okavango Delta water pans reveal novel insights into wildlife disease potential

**DOI:** 10.1002/imo2.70018

**Published:** 2025-04-29

**Authors:** Emilie J. Skoog, Kenosi Kebabonye, Benjamin Klempay, Mangaliso Gondwe, Kaelo Makati, Nlingisisi Babayani, Mosimanegape Jongman, Jeff Bowman, Lihini Aluwihare

**Affiliations:** ^1^ Scripps Institution of Oceanography University of California San Diego San Diego California USA; ^2^ Okavango Research Institute University of Botswana Maun Botswana; ^3^ Department of Biological Sciences University of Botswana Gaborone Botswana

## Abstract

Botswana's Seronga region saw a mass elephant die‐off potentially linked to water sources. This study analyzes Okavango Delta metagenomes, uncovering a diversity of viruses and harmful pathogens. Findings highlight the importance of understanding viral ecology in these waters and support One Health's objective in protecting human, animal, and ecosystem health.

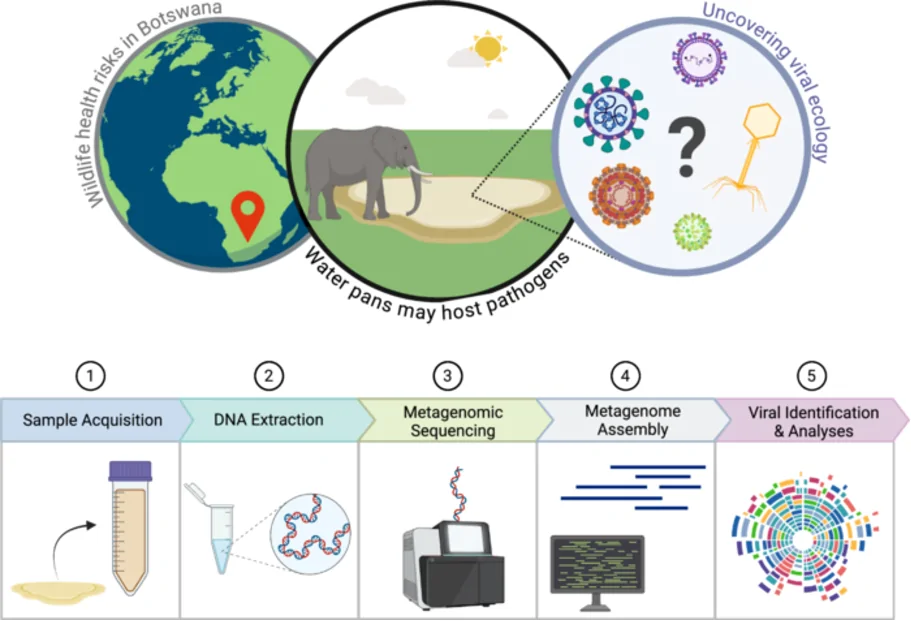

To the Editor,

The mass mortality of the African savannah elephants (*Loxodonta africana*) in Botswana in 2020 sparked widespread concern about wildlife disease outbreaks, with more than 350 deaths reported within 2 months in a relatively restricted area north of the Okavango Delta Panhandle region [1, 2, 3]. Early reports linked mortality to water sources because many of the carcasses were localized to seasonal water pans, but it became evident that little was known about the water quality and ecology of the water pans in this region [4]. To address this gap and provide baseline data for the water pans critical for supporting wildlife, we examined the viral ecology of three seasonal water pans in a remote region of the Okavango Delta panhandle for the first time. Our goal was to use the viral community composition of these water pans to better understand the risk that their ecology can impose on elephants and other wildlife.

Here we report the first data on the viral ecology of water pans in Seronga based on viruses assembled from water pan metagenomes. The viral communities are diverse and interestingly include poxvirus and EEHVs, both known to cause disease and even death among elephant species. Additionally, several viral auxiliary metabolic genes (AMGs) identified within the water pan viral metagenomes suggest the presence of additional mammalian disease‐causing agents. Human betaherpesviruses were also identified, indicating that these viral communities could pose an as‐yet unquantified risk to human health. While we do not suggest a direct connection between water pan viral content and known elephant or human disease outbreaks, by studying the overall ecology of these water pans, we further support the United Nations' One Health sustainability goal to address and optimize health threats at the animal‐human‐environment interface.

## 1 RESULTS AND DISCUSSION

**FIGURE 1:**
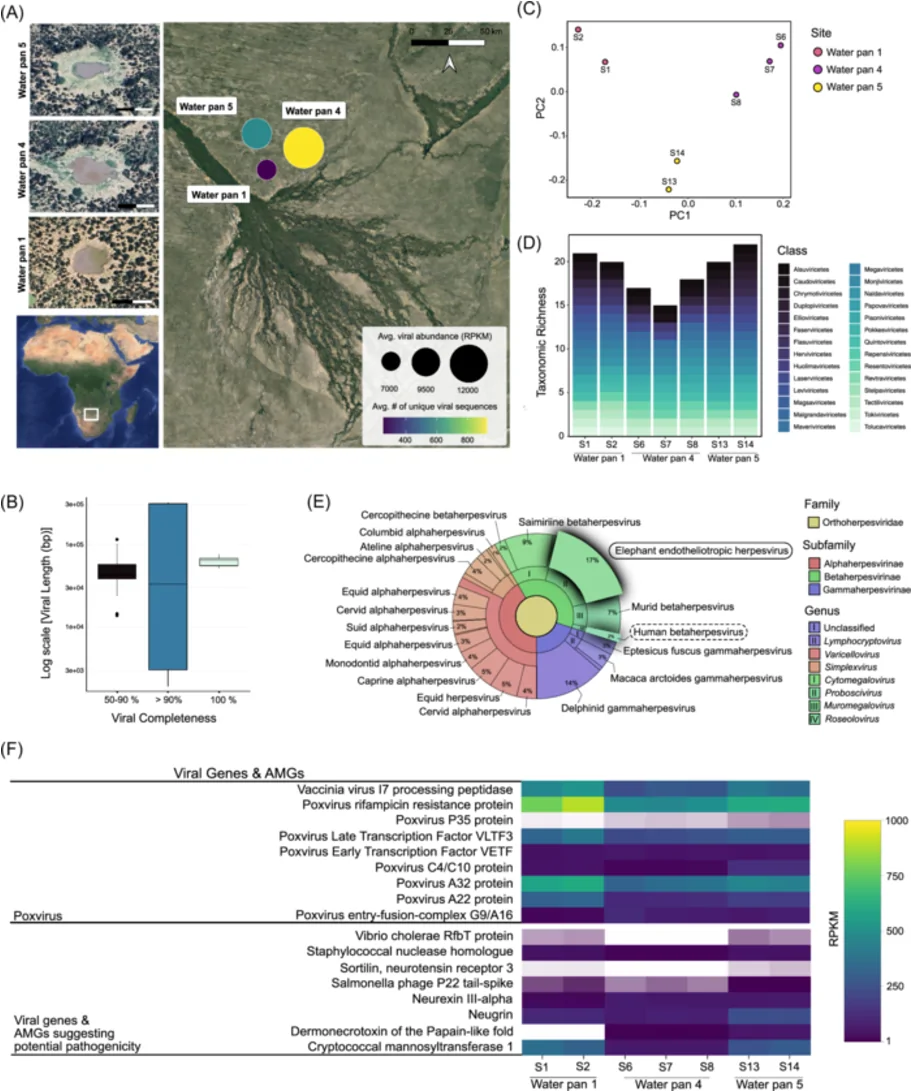
Viral presence and diversity in the Okavango Delta. (A) Map of the Okavango Delta showing the average number of unique, quality‐filtered viral sequences detected across samples from each water pan (point color) and the total normalized (RPKM) viral read values (point size) averaged across sites. (B) Summary of completeness and genome size (bp) for all viruses identified in Water Pan 1, Water Pan 4, and Water Pan 5. (C) Principal Coordinates Analysis (PCoA) plot illustrating beta diversity based on the Jaccard Index at the family level, showing clustering patterns of viral samples across sites. (D) Viral richness at the class level, highlighting the number of unique viral classes detected across water pan sites. (E) Krona chart showing the taxonomic identification and relative abundance of viruses in the *Orthoherpesviridae* family from representative samples across Water pan 5, including elephant (solid outline) and human (dotted outline) herpesviruses. (F) Heatmap showing the total normalized (RPKM) gene reads of potentially pathogenic viruses and genes identified within viral sequences from each water pan. White indicates the absence of the gene.

Seven water samples were collected from three water pans during the 2022 dry season: Water pan 1 (WP1; drying, turbid), Water pan 4 (WP4; moderately full), and Water pan 5 (WP5; moderately full) (Figure 1A; Table S1). Water samples were sequenced, assembled into seven metagenomes, and analyzed to investigate viral ecology. Although viral genome abundances derived from mixed‐community metagenomes obtained from the >0.22 µm size fraction may not accurately reflect viral abundances observed in viromes (<0.22 µm fraction), metagenomes can effectively identify numerous viral genomes, including those sometimes not detected in viromes [5]. Moreover, comparative analyses between viruses identified within viromes and metagenomes in freshwater environmental samples have demonstrated that there is no significant difference in the number of viral genomes or viral species richness between viromes and metagenomes in freshwater environments [5]. Therefore, although viral genomes obtained from viromes may be more ideal for viral analyses, using metagenomes assembled from these freshwater water pans remains a robust approach for investigating the viral ecology of this underexplored environment.

### 1.1 Viral ecology

Viruses within the metagenomic fraction of each of the three water pans were identified and quantified from quality‐filtered viral sequences at each site. Of the 92 medium‐to‐high quality viral genomes detected, ~7% were 100% complete, ~5% were 90%–99% complete, and ~88% were 50%–90% complete (Figure 1B). The highest averaged abundance (*n* = 12,190) of viral sequences was found in WP4, while WP1 possessed the lowest averaged viral abundance (*n* = 7014) within the metagenomic fraction (Figure 1A; Table S1). WP4 also exhibited the highest quantity of unique viral sequences (*n* = 876) and WP1 possessed the smallest number of unique viral genomes (*n* = 258; Figure 1A; Table S1). Beta diversity analyses of viral taxonomic communities at the family level revealed that viral communities from samples within the same water pan shared similar taxonomic profiles (Figure 1C).

At the class level, WP1 and WP5 exhibit the highest richness, with viruses classified into as many as 22 unique classes (Figure 1D). Taxonomies included dsDNA viruses (i.e., Caudoviricetes, Naldaviricetes, Papovaviricetes, Pokkesviricetes, Tokiviricetes), ssDNA viruses (i.e., Malgrandaviricetes, Repensiviricetes, Faserviricetes, Quintoviricetes), large DNA viruses (i.e., Megaviricetes), and virophage (i.e., Maveriviricetes) capable of infecting a large array of organisms including prokaryotes and eukaryotes. RNA viruses were also identified—likely due to the happenstance capture of reverse‐transcribed viral RNA within the metagenomes—and included ssRNA viruses (i.e., Leviviricetes, Quintoviricetes), +ssRNA viruses (i.e., Alsuviricetes, Magsaviricetes, Pisoniviricetes, Stelpaviricetes, Tolucaviricetes), −ssRNA viruses (i.e., Ellioviricetes, Monjiviricetes), ssRNA‐RT retroviruses (i.e., Revtraviricetes), and dsRNA viruses (i.e., Chrysmotiviricetes, Duplopiviricetes, Resentoviricetes). The presence of viruses classified within the Huolimaviricetes, Faserviricetes, and Duplopiviricetes classes was unique to WP5. Viruses of the Stelpaviricetes, Tolucaviricetes, Magsaviricetes, and Flasuviricetes classes were only identified at WP1. Chrymotiviricetes was unique to WP1 and WP5, and Resentoviricetes was only identified at WP1 and WP4. Papovaviricetes and Repensiviricetes were only present at WP4 and WP5.

A diverse array of viruses, capable of infecting a broad range of hosts, was detected across all water pans. Viruses infecting archaea, such as Tokiviricetes and Huolimaviricetes, and those targeting bacteria, including Caudoviricetes, Malgrandaviricetes, and Leviviricetes, were identified within water pans. Other detected classes, including Repensiviricetes, Tolucaviricetes, and Monjiviricetes, have been reported to infect fungi and plants [6]. Notably, several sequenced viral classes including Herviviricetes, Pokkesviricetes, Stelpaviricetes, Papovaviricetes, Monjiviricetes, Ellioviricetes, and Quintoviricetes are capable of infecting a diverse range of wildlife [7].

### 1.2 Distinct mammal‐associated viral communities

Among the identified viral communities, the Pokkesviricetes class includes viruses belonging to the *Poxviridae* family, which is comprised of members of the poxvirus species, several of which are known to infect humans and elephants among other hosts [8, 9, 10]. Additionally, the Herviviricetes class includes the *Orthoherpesviridae* family, which consists of viruses known to infect animals, including humans and elephants. Notably, *Orthoherpesviridae* viruses were detected in each water pan sampled. Across all water pans, up to 17% of identified viruses belonging to the *Orthoherpesviridae* family were identified as elephant endotheliotrophic herpesviruses (EEHVs), known to cause hemorrhagic disease and often sudden death in both Asian and African elephant species (Figure 1E; Figure S1; [11]). Although these metagenome samples are not directly connected to mass elephant mortality events, these findings do suggest the potential presence of viruses that may target elephants and other mammalian wildlife. This underscores the importance of understanding baseline viral communities present in these water pans, which serve as critical water sources for wildlife survival. Members of this viral community may also be benign components of the wildlife microbiome until animals are exposed to environmental stresses such as water and food scarcity, extreme temperatures, and wildfires, which are increasing threats due to climate change and anthropogenic activities in arid regions [12]. Additionally, sequencing of the viral community also revealed the presence of human betaherpesvirus—up to 11% of viruses belonging to the *Orthoherpesviridae* family—in each of the water pans, which could pose a direct threat to human health (Figure 1E; Figure S1; [13]).

### 1.3 Viral AMGs, and potential pathogenicity

To better understand potential viral pathogenicity at each of these water pans, we annotated each viral sequence and identified viral genes and viral AMGs that suggest potential virulence. Several poxvirus‐related genes were detected in each water pan (Figure 1F), confirming the presence of poxvirus (class Pokkesviricetes) in these waters. This virus has been associated with fatal skin and mucosal lesions in elephants, humans, and other mammals globally [8, 9, 10]. Interestingly, a dermonecrotoxin gene, a major virulence factor produced by certain strains of *Pasteurella multocida* and known to cause ulceration and dermal necrosis, was also identified in viral sequences from WP4 and WP5 (Figure 1F; [14]). Notably, close relatives of *Pasteurella multocida* have been implicated as potential contributors to several elephant deaths in Zimbabwe during the same period in 2020 [15]. Other identified viral AMGs including sortilin (neurotensin receptor 3), neugrin, and neurexin III‐alpha (NRXN3) are known to play key roles in mammalian neurological function [16, 17, 18]. This is particularly intriguing given the hypothesis that the mass elephant mortality event in Botswana may have been linked to neurological symptoms [1]. Additionally, genes associated with large eukaryotic DNA viruses, capable of infecting animals such as elephants and humans, were detected across the water pans, with the highest abundance observed in WP1 (Figure S2). Several toxin, anti‐toxin, and antimicrobial AMGs reveal an additional diversity of genes that could influence the ecological pathogenicity of these water pans (Figure S2).

Results support the vast diversity of viruses in these water pans and reveal their potential influence on the wildlife that depend on these water sources. Although the number of water pan samples in the current study is limited, this study provides an important first glimpse into their viral ecology and highlights the need for future ecological assessment that includes a broader range of water pans. Furthermore, seasonal fluctuations, particularly drying and rewetting cycles, likely play a significant role in shaping viral communities and concentration in water pans. In particular, dry season sampling is especially important, as this is when wildlife pressure is highest and pans are most heavily used, potentially concentrating viral particles and increasing the likelihood of wildlife‐mediated transmission of potential viral pathogens. Future studies that incorporate seasonal sampling will be critical to understanding these dynamics more fully. Additionally, while viral ecology presents one aspect of water pan influence on overall ecosystem health, future work should also integrate additional biological and environmental factors (e.g., water quality, microbial pathogens, seasonal variations, etc.) to comprehensively assess ecosystem health.

### 1.4 Implications for the World Health Organization and One Health

Pastoralism is widely practiced in Botswana, meaning that in addition to wildlife health, these pathogens could additionally impact the livestock and people who interact with these water sources and potentially infected wildlife [19]. Wildlife disease outbreaks, such as those caused by poxvirus or EEHV, for example, pose significant challenges to the One Health initiative [15]. Studies have demonstrated that poxviruses can be transmitted from rodents to elephants, with the potential for subsequent transmission to humans [20]. Humans could also contract illnesses from water pans containing human‐infecting viruses, such as poxviruses and human betaherpesviruses, which were identified in this study. Although data on human interactions with these water pans are limited, there is evidence that livestock frequently visit them, which may have indirect implications for human health. Studying the viral ecology of these water pans provides a noninvasive approach to advancing the One Health sustainability goal by offering a more comprehensive method to establish baseline ecosystem health and assess and optimize the interconnected health of people, animals, and the environment, in addition to being a potent early warning approach to potential reemerging and emerging diseases.

## 2 METHODS

Detailed procedures for sample collection and viral metagenomic analyses are available in the Supplementary Information.

## AUTHOR CONTRIBUTIONS

**Emilie J. Skoog:** Data curation; formal analysis; visualization; writing—original draft, methodology, writing—review & editing. **Kenosi Kebabonye:** Writing—original draft; data curation; investigation; writing—review & editing. **Benjamin Klempay:** Data curation. **Mangaliso Gondwe:** Conceptualization; investigation; writing—review & editing. **Kaelo Makati:** Investigation; writing—review & editing. **Nlingisisi Babayani:** Conceptualization; writing—review & editing. **Mosimanegape Jongman:** Conceptualization; investigation; writing—review & editing. **Jeff Bowman:** Writing—review & editing; methodology; formal analysis; data curation. **Lihini Aluwihare:** Conceptualization; investigation; supervision; writing—review & editing; funding acquisition; project administration.

## CONFLICT OF INTEREST STATEMENT

The authors declare no conflicts of interest.

## 1 ETHICS STATEMENT

No animals or humans were involved in this study.

## Supporting information


**Figure S1.** Viruses from the *Orthoherpesviridae* family detected in water pan viral communities.
**Figure S2.** Potential viral taxonomies and associated pathogenic genes identified in viral communities across water pans.

**Table S1.** Site‐specific viral abundance values for each water pan sample location.

## Data Availability

The data that supports the findings of this study are available in the supplementary material of this article. Sequence data for metagenomics reads from each of the seven water pans can be found under NCBI BioProject ID PRJNA1167433 with accession numbers SAMN44049353 ‐ SAMN44049359 (https://www.ncbi.nlm.nih.gov/bioproject/?term=PRJNA1167433). The data and scripts used are saved in GitHub https://github.com/emilieskoog/Viral_metagenomics_Botswana_waterpans. Supplementary materials (methods, figures, tables, graphical abstract, slides, videos, Chinese translated version, and update materials) may be found in the online DOI or iMetaOmics http://www.imeta.science/imetaomics/.
