# Supplementary material for: Viral metagenomics of Okavango Delta water pans reveal novel insights into wildlife disease potential
Source: IMetaOmics. 2025 Apr 29;2(2):e70018. doi: 10.1002/imo2.70018 (PMC12806373; doi:10.1002/imo2.70018)
Supplement: Supplementary file 1 — Figure S1. Viruses from the Orthoherpesviridae family detected in water pan viral communities. Figure S2. Potential viral taxonomies and associated pathogenic genes identified in viral communities across water pans. [file IMO2-2-e70018-s002.docx]

**Supporting information to**

# Viral Metagenomics of Okavango Delta Water Pans Reveal Novel Insights into Wildlife Disease Potential

**Running title:** Viral Ecology of the Okavango Delta Water Pans

Emilie J. Skoog^1*#^, Kenosi Kebabonye^1#^, Benjamin Klempay^1^, Mangaliso Gondwe^2^, Kaelo Makati^2^, Nlingisisi Babayani^2^, Mosimanegape Jongman^3^, Jeff Bowman^1^, Lihini Aluwihare^1^

^1^*Scripps Institution of Oceanography, University of California San Diego, San Diego, CA, 92037, USA*

^2^*Okavango Research Institute, University of Botswana, Maun, Botswana*

^3^*Department of Biological Sciences, University of Botswana, Gaborone, Botswana*

^#^These authors contributed equally: Emilie J. Skoog, Kenosi Kebabonye

*Correspondence: [eskoog@ucsd.edu](mailto:eskoog@ucsd.edu) (Emilie J. Skoog)

**Graphical Abstract**

In 2020, the Seronga region adjacent to the Okavango Panhandle in Botswana experienced an unprecedented elephant mass-mortality event. This event remains unexplained; however, many carcasses were located near seasonal water pans raising questions about a potential link between wildlife health and drinking water. To collect baseline data on pathogen load in seasonal water pans, metagenomes were assembled from water collected from three pans located in the Okavango panhandle. Here we report the first data on the viral ecology of water pans in Seronga based on viruses assembled from water pan metagenomes. The viral communities are diverse and include viruses known to cause disease among elephant species and humans. Findings highlight the importance of understanding viral ecology in these waters, support the World Health Organization’s One Health initiative by promoting the integrated health of people, animals, and ecosystems, and enhance our ability to predict and mitigate emerging infectious diseases through environmental surveillance.

**MATERIALS AND METHODS**

**Sample Collection**

In July of 2022, seven water samples were collected across three water pans in the panhandle of the Okavango Delta: Water pan 1, Water pan 4, Water pan 5 (Fig. 1; Table S1) under permit ENT 8/36/4L(93). 3-9 L of water was collected from each water pan. Water samples were pre-filtered through sieves with pore sizes of 23 µm to remove sediment particles. However, this pre-filtering step proved insufficient in removing fine particulates, as evidenced by the presence of minimal material exceeding 3 µm in size. To remove fine sediment particulates for subsequent filtration, each sample was centrifuged at 5000 rpm for 20 minutes at 4°C in 50 mL sterile Falcon tubes. The supernatant was then filtered onto 0.22 µm, 47 mm Pall Supor membrane, gamma sterilized, gridded filters. Filters were subsequently transferred into 3 mL, sterile cryovials, and placed immediately in a liquid nitrogen charged dry shipper. Filters remained in the cold dry shipper until they were transferred to a -80°C freezer and stored until nucleic acid extraction.

**DNA Extraction and Purification**

DNA was extracted from one quarter of each 0.22 µm, 47 mm filter using the MagMAX Microbiome Ultra Nucleic Acid Isolation Kit (ThermoFisher Scientific, Waltham, MA, USA) with the KingFisher Flex high-throughput extraction system (ThermoFisher Scientific). DNA concentrations were quantified using the Qubit dsDNA High Sensitivity Assay (Invitrogen, Waltham, MA, USA). Replicates which yielded <1 ng µL-1 extracted DNA were pooled by sample and concentrated using Amicon Ultra-0.5 mL Centrifugal Filters (Millipore, Burlington, MA, USA) with 3 kDa cutoff by spinning for the maximum time (30 min) at 14,000 × g. For samples which yielded >1 ng µL-1 extracted DNA, the replicate with the highest yield was selected (without concentration) for Whole Metagenome Shotgun (WMGS) sequencing.

**Metagenomic Sequencing and Assembly**

Selected combined and concentrated samples were submitted to the UC San Diego Microbiome Core (La Jolla, CA) for library prep using the KAPA HyperPlus Kit (Roche Sequencing Solutions, Basel, Switzerland) and WMGS sequencing with the Illumina NovaSeq 6000 platform. Raw metagenomic sequencing reads were pre-processed using a customized version of the iMAGine pipeline [1].  Briefly, reads were QC’d with fastp v0.23.2 [2] to a minimum score of 30.  QC’d reads were assembled using metaSPAdes v3.15.5 [3] and k-mer lengths of 21, 33, and 55 bases. Reads from all libraries were mapped to contigs with BWA [4] and further processed with SAMtools [5]. Contigs with a minimum length of 1500 bases were binned using MetaBAT2 [6] and the completion and contamination of the resulting bins assessed with CheckM [7].

**Viral Sequence Identification and Taxonomic Classification**

Viral reads were taxonomically classified using Kraken2 v2.1.2 [8], a reference database-based tool used to identify and classify viral communities. In addition to using Kraken2 for viral identification, geNomad [9] – a machine learning-based approach specifically optimized for detecting novel viral sequences in metagenomic data – was also employed, enabling the identification of viral sequences that may not be detectable through reference database-based approaches. GeNomad v1.5.2 [9] was used with default parameters to predict viral sequences from the metagenomic assemblies obtained from Water pan 1, Water pan 4, and Water pan 5. Putative viral sequences were quality filtered, and host regions were removed from all viral contigs using CheckV v1.0.3 (database v1.5; [10]). Sequences where CheckV was not able to resolve viral quality (i.e., “not-determined”) were excluded from analysis. Trimmed viral and prophage sequences that contained no viral genes, were smaller than 10 kbp, and had predicted values of greater than 10% contamination were excluded from further analysis.

**Viral Diversity Analyses**

Alpha diversity was assessed by calculating taxonomic richness across samples at the class level and visualized using ggplot2 v3.5.1 [11]. To assess beta diversity, the Jaccard Index was calculated and used to quantify the compositional dissimilarity between samples based on presence/absence of taxa at the family level. Pairwise Jaccard dissimilarity values were computed using the vegdist() function from the vegan v2.6-8 package [12] in R v4.4.2. Additionally, viral reads outputted by Kraken2 were visualized using KronaTools v2.7 [13] to explore hierarchical metagenomic data (ktImportTaxonomy function) and create interactive charts displaying the relative abundance of microbial and viral taxa across samples.

**Presence and Relative Abundance of Viral Auxiliary Metabolic Genes (AMGs)**

Viral sequences were functionally annotated via alignment to the Pfam-A [14], TIGRFAM [15], KEGG Orthology [16] and COG databases [17] as part of the geNomad v1.5.2 pipeline [9]. This annotation process included the identification of auxiliary metabolic genes (AMGs), which were manually curated to ensure accuracy. These AMGs were then categorized into broader functional categories, reflecting their roles in viral and host metabolism and association. To assess relative abundance of viral AMGs, quality-filtered viral reads identified from each of the seven samples across the three water pan sites were pooled and collectively indexed and mapped against each of the seven metagenomes using Bowtie2 v2.4.2 [18], utilizing the flags --no-unal --very-sensitive. SAMtools v1.12 [5] was subsequently used to create and sort BAM files from the generated SAM files. Normalized relative abundance of each viral sequence at each water pan was calculated utilizing the Reads Per Kilobase Million (RPKM) method with CoverM (v0.7.0) [19] using contig --min-read-percent-identity 0.95 --min-covered-fraction 0.5 parameters.

**SUPPLEMENTAL FIGURE AND TABLE LEGENDS**

**Figure S1.** **Viruses from the *Orthoherpesviridae* family detected in water pan viral communities.** Krona charts showing the taxonomic identification and relative abundance of viruses in the *Orthoherpesviridae* family from representative samples across A) Water pan 1, B) Water pan 4, and C) Water pan 5, including elephant (solid outline) and human (dotted outline) herpesviruses.

**Figure S2.** **Potential viral taxonomies and associated pathogenic genes identified in viral communities across water pans.** Heatmap showing the total normalized (RPKM) gene reads of virus-associated and potentially pathogenic genes identified within viral sequences from each water pan. White indicates the absence of the gene.

**REFERENCES**

1. Dutta, Avishek, Elizabeth Connors, Rebecca Trinh, Natalia Erazo, Srishti Dasarathy, Hugh W. Ducklow, Deborah K. Steinberg, Oscar M. Schofield, and Jeff S. Bowman. 2023. “Depth drives the distribution of microbial ecological functions in the coastal western Antarctic Peninsula.” *Frontiers in Microbiology* **14**: 1168507. https://doi.org/10.3389/fmicb.2023.1168507

2. Chen, Shifu, Yanqing Zhou, Yaru Chen, and Jia Gu. 2018. “fastp: an ultra-fast all-in-one FASTQ preprocessor.” *Bioinformatics* **34**: i884–i890. https://doi.org/10.1093/bioinformatics/bty560

3. Nurk, Sergey, Dmitry Meleshko, Anton Korobeynikov, and Pavel A. Pevzner. 2017. “metaSPAdes: a new versatile metagenomic assembler.” *Genome Research* **27**: 824–834. https://doi.org/10.1101/gr.213959.116

4. Houtgast, Ernst Joachim, Vlad-Mihai Sima, Koen Bertels, and Zaid Al-Ars. 2018. “Hardware acceleration of BWA-MEM genomic short read mapping for longer read lengths.” *Computational Biology and Chemistry* **75**: 54–64. https://doi.org/10.1016/j.compbiolchem.2018.03.024

5. Danecek, Petr, James K. Bonfield, Jennifer Liddle, John Marshall, Valeriu Ohan, Martin O. Pollard, Andrew Whitwham, et al. 2021. “Twelve years of SAMtools and BCFtools.” *Gigascience* **10**: giab008. https://doi.org/10.1093/gigascience/giab008

6. Kang, Dongwan D., Feng Li, Edward Kirton, Ashleigh Thomas, Rob Egan, Hong An, and Zhong Wang. 2019. “MetaBAT 2: an adaptive binning algorithm for robust and efficient genome reconstruction from metagenome assemblies.” *PeerJ* **7**: e7359. https://doi.org/10.7717/peerj.7359

7. Parks, Donovan H., Michael Imelfort, Connor T. Skennerton, Philip Hugenholtz, and Gene W. Tyson. 2015. “CheckM: assessing the quality of microbial genomes recovered from isolates, single cells, and metagenomes.” *Genome Research* **25**: 1043–1055. https://doi.org/10.1101/gr.186072.114

8. Wood, Derrick E., Jennifer Lu, and Ben Langmead. 2019. “Improved metagenomic analysis with Kraken 2.” *Genome Biology* **20**: 1–13. https://doi.org/10.1186/s13059-019-1891-0

9.Camargo, Antonio Pedro, Simon Roux, Frederik Schulz, Michal Babinski, Yan Xu, Bin Hu, Patrick SG Chain, Stephen Nayfach, and Nikos C. Kyrpides. 2024. “Identification of mobile genetic elements with geNomad.” *Nature Biotechnology* **42**: 1303–1312. https://doi.org/10.1038/s41587-023-01953-y

10. Nayfach, Stephen, Antonio Pedro Camargo, Frederik Schulz, Emiley Eloe-Fadrosh, Simon Roux, and Nikos C. Kyrpides. 2021. “CheckV assesses the quality and completeness of metagenome-assembled viral genomes.” *Nature Biotechnology* **39**: 578–585. https://doi.org/10.1038/s41587-020-00774-7

11. Wickham, Hadley. 2016. “ggplot2: Elegant Graphics for Data Analysis.” https://ggplot2.tidyverse.org

12. Oksanen, Jari, F. Guillaume Blanchet, Roeland Kindt, Pierre Legendre, Peter R. Minchin, R. B. O’hara, Gavin L. Simpson, et al. 2022. “Package ‘vegan’.” *Community Ecology Package* **2**: 1–295. https://github.com/vegandevs/vegan

13. Ondov, Brian D., Nicholas H. Bergman, and Adam M. Phillippy. 2011. “Interactive metagenomic visualization in a Web browser.” *BMC Bioinformatics* **12**: 1–10. https://doi.org/10.1186/1471-2105-12-385

14. Mistry, Jaina, Sara Chuguransky, Lowri Williams, Matloob Qureshi, Gustavo A. Salazar, Erik LL Sonnhammer, Silvio CE Tosatto, et al. 2021. “Pfam: The protein families database in 2021.” *Nucleic Acids Research* **49**: D412–D419. https://doi.org/10.1093/nar/gkaa913

15. Haft, Daniel H., Jeremy D. Selengut, and Owen White. 2003. “The TIGRFAMs database of protein families.” *Nucleic Acids Research* **31**: 371–373. https://doi.org/10.1093/nar/gkg128

16. Kanehisa, Minoru, and Susumu Goto. 2000. “KEGG: kyoto encyclopedia of genes and genomes.” *Nucleic Acids Research* **28**: 27–30. https://doi.org/10.1093/nar/28.1.27

17. Galperin, Michael Y., Yuri I. Wolf, Kira S. Makarova, Roberto Vera Alvarez, David Landsman, and Eugene V. Koonin. 2021. “COG database update: focus on microbial diversity, model organisms, and widespread pathogens.” *Nucleic Acids Research* **49**: D274–D281. https://doi.org/10.1093/nar/gkaa1018

18. Langmead, Ben, and Steven L. Salzberg. 2012. “Fast gapped-read alignment with Bowtie 2.” *Nature Methods* **9**: 357–359.

19. Aroney, Samuel TN, Rhys JP Newell, Jakob N. Nissen, Antonio Pedro Camargo, Gene W. Tyson, and Ben J. Woodcroft. 2025. “CoverM: Read alignment statistics for metagenomics.” *arXiv preprint arXiv:2501.11217*. https://doi.org/10.48550/arXiv.2501.11217
